# Lack of educational impact of video game addiction in children and adolescents diagnosed with ADHD: A cross-sectional study

**DOI:** 10.3389/fpsyt.2023.1136671

**Published:** 2023-04-20

**Authors:** Hilario Blasco-Fontecilla, Ángela Menéndez-García, Fernando Sanchez-Sanchez, Marcos Bella-Fernández

**Affiliations:** ^1^Faculty of Medicine, Autonomous University of Madrid, Madrid, Spain; ^2^Department of Psychiatry, IDIPHISA-Puerta de Hierro University Hospital, Majadahonda, Spain; ^3^Spain Biomedical Research Networking Center for Mental Health Network (CIBERSAM), Madrid, Spain; ^4^Korian, ITA Salud Mental, Madrid, Spain; ^5^R&D Department, TEA Ediciones, Madrid, Spain; ^6^Faculty of Psychology, Autonomous University of Madrid, Madrid, Spain; ^7^Department of Psychology, Pontifical University of Comillas, Madrid, Spain

**Keywords:** attention deficit and hyperactivity disorder, internet gaming disorder, academic performance, children, adolescents

## Abstract

**Introduction:**

The use of video games has become widespread worldwide. Excessive use of video games is increasingly becoming a matter of concern, particularly in patients diagnosed with Attention Deficit Hyperactivity Disorder (ADHD). Currently, internet gaming disorder (IGD) is not included within the DSM-5-chapter *Disorders related to substances and addictive disorders*.

**Methods:**

This is a *post-hoc* descriptive naturalistic study comparing children and adolescents diagnosed with ADHD with and without IGD. We used the 85% cut-off point of the test ADITEC-V for video game addiction to split our sample of ADHD patients into those with IGD (>=85%) and those without IGD (<85%).

**Results:**

13 (25%) out of the 51 children and adolescents with ADHD included in our study had an IGD. Patients with IGD had a first contact with internet, smartphones, and videogames at a very early age (5.67 ± 3.31, 6.33 ± 4.60, and 7.50 ± 2.61, respectively). However, only age at first contact with the internet was statistically significantly different when comparing ADHD patients with and without IGD (8.68 ± 2.71 vs. 5.67 ± 3.31, *t* = 3.166, df = 47, *p* = 0.01). Different neurodevelopmental, clinical, and neuropsychological measures converging in impulsivity is a risk factor for IGD. Unexpectedly, we found no association between IGD and poor academic achievement.

**Discussion:**

Future studies may include randomized controlled trials for treating IGD, the study of social adjustment as a protective factor against developing an IGD, and the role of serious and non-serious video games in the development of an IGD, among others. Additional research is clearly needed on IGD.

## Introduction

With the advent of new technologies, the use of video games has become widespread worldwide. Excessive use of video games is increasingly becoming a matter of concern. For instance, 15% of young people spend three or more hours playing video games on a school day (1). Indeed, there is increasing evidence that “Internet Gaming Disorder” (IGD) can be considered a behavioral addiction (2–4). The Diagnostic and Statistical Manual of Mental Disorders, fifth edition (DSM-5) did not include IGD within the chapter *Disorders related to substances and addictive disorders*, but expressed the current concern by including IGD as a condition for further study in Section 3 (4).

These are several risk factors for IGD, including psychological impairments such as impulsivity (5), attention problems or emotional dysregulation (6), as well as lack of social and interpersonal skills (6, 7). In turn, Attention Deficit Hyperactivity Disorder (ADHD), the most frequent neurodevelopmental disorder in childhood and adolescence with a prevalence of 5% worldwide (8–10), is related to impulsivity, attention difficulties and emotional dysregulation (11–13), among others. The association between ADHD and IGD has been increasingly studied in recent years. Some studies reported no significant differences in the amount of time or frequency of play between children with and without ADHD, but rather a more severe addiction to IGD in children with ADHD (14). In a similar vein, other studies have found that adolescents with ADHD and IGD have more severe clinical ADHD, greater socio-emotional impairment, greater withdrawal symptoms (15), and greater difficulties in recovery than patients with IGD but not ADHD (16). In a previous study using the cases (with ADHD) and controls (without ADHD) in this sample, we found that the severity of hyperactivity/impulsivity symptoms and IGD were moderately correlated (r = 0.349, *p* = 0.013), but the correlation disappeared after controlling for the social domain. In other words, good social adaptation buffered the relationship between hyperactivity and IGD (17).

The main aim of this study is to explore whether or not children and adolescents with ADHD and IGD as measured by the ADITEC Video Games (ADITEC-V) Questionnaire (18) display a differential profile compared to children and adolescents with ADHD but not IGD. We hypothesized that: (1) hyperactivity/impulsivity measures are associated with IGD; and (2) children and adolescents with ADHD and IGD have poorer academic achievement than children and adolescents with ADHD but not IGD.

## Methods

### Participants

Our study is a *post-hoc* descriptive naturalistic study comparing children and adolescents diagnosed with ADHD with and without IGD (17). The sample was recruited at the Puerta de Hierro University Hospital in Majadahonda (HUPH-M). Fifty-one children and adolescents diagnosed with ADHD between 7 and 17 years old were recruited. None of the patients presented comorbidity with mental retardation, generalized developmental disorders or other neurological or psychiatric alterations that could compromise the cognitive functioning of the participant.

### Measures and procedure

All patients were evaluated using semi-structured diagnostic interviews, including parent interviews, and a protocol including sociodemographic and clinical data; the ADHD criteria of the Diagnostic and Statistical Manual of Mental Disorders, 4th ed. (DSM-IV); and some scales for assessing ADHD and IGD.

ADHD patients were divided into: (1) predominantly hyperactive/impulsive type (ADHD/HI) or combined type (ADHD/C), and (2) predominantly inattentive type (ADHD/I). To evaluate the main ADHD symptoms (impulsivity, hyperactivity and inattention), we used the Spanish version of the Swanson, Nolan and Pelham Scale for parents (SNAP-IV) (19, 20), and the Abbreviated Conner’s Rating Scales for parents (CPRS-HI) (21). We used raw scores for both scales. The SNAP-IV is one frequently used instrument for evaluating treatment response. The SNAP-IV consists of an 18-item checklist scored using a 4-point Likert scale (ranging from *Not at All* (0) to *Very Much* (3) [range 0 to 54]). The CPRS-HI is a 10-item checklist, a well-validated screening instrument for ADHD. This CPRS-HI consists of 10 behavioral statements rated on a 4-point Likert scale (global score ranging from 0 to 30). A score of 15 or higher is considered a good screening diagnostic cut-off point for diagnosing children with ADHD (22). Furthermore, we also have information regarding a set of different scales used routinely in the first evaluation of all patients. Thus, we had information regarding executive functioning as measured by the Behavior Rating Inventory of Executive Function (BRIEF) (23). The BRIEF is a standardized, validated instrument that assesses executive functioning, including 8 subscales comprising 2 indices and a Global Executive Composite (24). We also routinely included the Conners Continuous Performance Test, 3rd Edition (Conners CPT 3), which is a well-validated and standardized computerized go/no-go and attention test measuring attention and impulsivity (25). Results are described with different variables: correct hits (number of cases where a response occurs in the presence of a target); commission errors (number of cases where a response occurs in the presence of a non-target); mean reaction time (hit reaction time); and variability of hit reaction time (measured by standard deviation). These indicators are recorded for every block and group. Commission and omission errors measure impulsivity and inattention, respectively. Finally, results from an IQ test conducted in the last 18 months were available for some patients. The IQ information came mostly from the Wechsler Intelligence Scale for Children (WISC-IV) (26).

We evaluated the IGD using the ADITEC Video Games (ADITEC-V) Questionnaire, which provides information regarding compulsive gambling, tolerance abstinence, interference with other activities, and associated problems, and escape (Chóliz, Marco & Chóliz, 2016). We used the 85% cut-off point, which is different in females (> = 29) and males (> = 55) to split our sample of ADHD patients into those with IGD (> = 85%), and those without IGD (<85%). Furthermore, participating children’s parents filled out a 12-item (yes/no) questionnaire based on an adapted version of the criteria used for the evaluation of behavioral addictions (Blasco-Fontecilla et al., 2014). This strategy has been used elsewhere (Kourosh, Harrington & Adinoff, 2010).

Finally, we included information regarding some laboratory analyses routinely incorporated into the clinical history of ADHD patients in our consultation.

### Statistical analyses

We performed descriptive analyses of sociodemographic and clinical variables comparing those with ADHD and with or without IGD. We used χ2 for ordinal and internal variables, and the *t* test for independent samples associated with the Levene statistic for equality of variances to compare quantitative variables (ADHD with IGD vs. ADHD without IGD). The level of statistical significance was established at *p* < 0.05. After applying Bonferroni correction (55 comparisons), the final significance was *p* < 0.001. We used the SPSS software (v20 for Mac) for all analyses.

### Ethics statement

The present study was carried out in accordance with the Declaration of Helsinki. This study was approved by the local Institutional Review Board (February 12, 2018; n° 03.18). Before entering the study, both the parents (or legal guardians) and the children were adequately informed, and agreed to participate voluntarily. Informed Consent (IC) was obtained from both parents (or legal guardians) and the children.

## Results

Thirteen (25%) out of the 51 children and adolescents with ADHD included in our study had an IGD. Children and adolescents with ADHD and IGD had a first contact with internet, smartphones and video games at a very early age (5.67 ± 3.31, 6.33 ± 4.60, and 7.50 ± 2.61, respectively). Tables 1 2 display the participants’ sociodemographic and neurodevelopmental characteristics, and some medical and ADHD characteristics, including some results from blood analyses, respectively.

**Table 1:** Patient sociodemographic and developmental characteristics.

	No IGD (*n* = 38) %	IGD (*n* = 13) %	Significance
Gender (Female)	18.4	30.8	FET* *p* = 0.439
Age	12.23 ± 2.71	11.11 ± 2.77	*t* = 1.279, df = 49, *p* = 0.207
Country (Spain)	81.6	84.6	FET *p* = 1
Ethnicity (Caucasian)	86.8	84.6	FET *p* = 0.643
Adopted (Yes)	13.5	23.1	FET *p* = 0.413
Problems during pregnancy (No)	81.2	55.6	FET *p* = 0.185
Gestational age < 37 weeks	15.8	**7.7**	FET *p* = 1
Problems during pregnancy (Yes)	10.3	28.6	FET *p* = 0.244
Low birth weight (<1.5 kg) (Yes)	2.7	0	FET *p* = 1
Time (months) lactating	9.63 ± 15.03	3.31 ± 0.96	*t* = 1.176, df = 32, *p* = 0.248
Problems during childbirth (Yes)	6	0	FET *p* = 1
Speech Acquisition (> 2 years old)	45.7	38.5	FET *p* = 0.750
Age of walking (First steps) (>1 years old)	82.4	41.7	**FET *p* = 0.021**
Full control urine sphincter >6 years old or older	19.4	25	FET *p* = 0.695
Full control anal sphincter >5 years old or older	5.9	8.3	FET *p* = 1

*FET, Fisher’s Exact Test. Significant differences in bold.

**Table 2:** Medical and ADHD antecedents, and blood analyses.

	No IGD (*n* = 38)%	IGD (*n* = 13)%	Significance
Familial antecedents of mental disorders	20.7	61.1	**FET *p* = 0.001**
Medical antecedents (Yes)	36.6	33.3	FET *p* = 1
ADHD Subtype (Hyperactive or mixed)	43.2	76.9	*FET p = 0.054*
Undergoing multimodal treatment (Drug + Psychotherapy)	38.9	69.2	FET *p* = 0.104
Time (years) on ADHD drug treatment	1.27 ± 1.4	1.46 ± 1.30	*t* = −0.416, df = 43, *p* = 0.68
Cholesterol	154 ± 30.27 (*n* = 19)	163.73 ± 29.45 (*n* = 11)	*t* = −0.856, df = 28, *p* = 0.40
Triglyceride	58.82 ± 28.34 (*n* = 17)	71.6 ± 35.2 (n = 11)	*t* = −1.034, df = 25, *p* = 0.311
Ferritin	41.29 ± 16.08 (n = 17)	29.67 ± 8.21 (*n* = 9)	*t* = 2.020, df = 24, *p* = 0.06
Vitamin D	75.93 ± 20.59 (*n* = 18)	55.95 ± 7.86 (*n* = 10)	*t = 2.932, df = 26, p = 0.01*
TSH	1.92 ± 0.8 (*n* = 18)	2.34 ± 0.76 (*n* = 10)	*t* = −1.343, df = 26, *p* = 0.191
% Eosinophils	4.98 ± 3.13 (*n* = 19)	4.88 ± 2.97 (*n* = 11)	*t* = −0.92, df = 28, *p* = 0.927
Ig E	246.76 ± 526.31 (*n* = 16)	798.65 ± 1379.73 (*n* = 10)	*t* = −1.454, df = 24, *p* = 0.25

FET: Fischer’s Exact Test. In italics, comparisons which reached significance before applying Bonferroni correction. In bold, comparisons which remained significant after Bonferroni correction.

We found that patients with IGD tended to suffer from a predominantly hyperactive ADHD subtype in a larger proportion than patients without IGD (see Table 2). Accordingly, we explored which neuropsychological characteristics could be involved. We found that the clinical scales and the CPT-3 commission parameters were related to IGD, giving further support to the clinical finding concerning the relationship between the hyperactive domain, and IGD (see Table 3). Table 3 summarizes the neuropsychological profile of ADHD patients with and without IGD.

**Table 3:** Neuropsychological profiles.

	No IGD (*n* = 38) (*n*)	IGD (*n* = 13) (*n*)	Significance
Intelligence quotient	104.36 ± 16.35 (22)	97.87 ± 19.57(8)	*t* = 0.913, df = 28, *p* = 0.37
*Verbal quotient*	104.09 ± 18.91 (22)	97.87 ± 19.57	*t* = 1.335, df = 16, *p* = 0.2
*Working memory*	102.56 ± 16.05 (9)	104.5 ± 7.94 (4)	*t* = −0.226, df = 11, *p* = 0.82
*Processing speed*	100 ± 10.44 (10)	90.2 ± 14.86 (5)	*t* = 1.494, df = 13, *p* = 0.16
*Perceptual reasoning*	107. 9 ± 18.86 (10)	88.5 ± 14.15 (4)	*t* = 1.842, df = 12, *p* = 0.09
SNAP-IV (inattention)	6.89 ± 2	6.4 ± 3.13	*t* = 0.581, df = 37, *p* = 0.56
SNAP-IV (hyperactivity)	3.96 ± 2.95	6.7 ± 2.54	*t* = *−2.106, df = 35, p = 0.01*
CPRS (screening)	13.7 ± 4.92	19.14 ± 6.82	*t = −2.280, df = 25, p = 0.03*
BRIEF global score	84.89 ± 31.96	110.2 ± 38.05	*t* = −1.518, df = 22, *p* = 0.14
CPT-3 response style	52.25 ± 9.93	60.28 ± 13.94	*t* = −1.946, dr = 38, *p =* 0.06
CPT-3 detectability	49.82 ± 8.5	48.28 ± 20.3	*t* = 0.482, df = 38, *p* = 0.63
CPT-3 omissions	50.7 ± 12.32	61.18 ± 25.18	*t* = −1.733, df=, *p* = 0.26
CPT-3 commissions	41.02 ± 5.3	48.22 ± 8.41	*t* = *2.418, df = 38, p = 0.01*
CPT-3 Perseverations	51.23 ± 10.29	48.95 ± 4.63	*t* = 0.641, df = 38, *p* = 0.52
CPT-3 HRT	57.64 ± 11.32	62.64 ± 18.53	*t* = −1.002, df = 38, *p* = 0.32
CPT-3 HRT standard deviation	52.5 ± 11.73	55.91 ± 14.99	*t* = −0.720, df = 38, *p* = 0.48
CPT-3 variability	49.89 ± 9.97	116.51 ± 191.57	*t* = −2.005, df = 37, *p* = 0.36
CPT-3 HRT block change	53.72 ± 9.72	47.89 ± 23.4	*t* = 1.110. df = 38, *p* = 0.27
CPT-3 HRT interstimulus interval change	53.3 ± 13.4	50. 61 ± 19.55	*t* = 0.477, df = 38, *p* = 0.636

BRIEF, Behavior Rating Inventory of Executive Function; CPRS, Conners’ Rating Scale for Parents; CPT-3, Conners’ Continuous Performance Test 3rd Edition; HRT, Hit Reaction Times; IGD, Internet Gaming Disorder; SNAP-IV, Swanson, Nolan and Pelham Scale for parents. In italics, comparisons which reached significance before applying Bonferroni correction. In bold, comparisons which remained significant after Bonferroni correction.

Table 4 deals with the pattern of gaming use and related issues (for instance, having holidays).

**Table 4:** Pattern of gaming use and related issues.

	No IGD % (*n*)	IGD % (*n*)	Significance
Age at first contact with video games	6.97 ± 3.39	7.50 ± 2.61	*t* = −0.491, df = 47, *p* = 0.58
Age at first contact with smartphones	7.30 ± 5.40	6.33 ± 4.60	*t* = 0.555, df = 47, *p* = 0.55
Age at first contact with internet	8.68 ± 2.71	5.67 ± 3.31	*t* = *3.166, df = 47, p = 0.01*
Hours a day (school days) social net	0.93 ± 2.60 (34)	2.59 ± 5.69 (12)	*t* = −1.363, df = 44, *p* = 0.348
Hours a day (weekend) social net	0.43 ± 1.13 (7)	4.25 ± 8.5 (4)	*t* = −1.221, df = 9, *p* = 0.44
Hours a day (school days) video games	0.86 ± 1.13 (35)	1.57 ± 1.43 (12)	*t* = *−1.722, df = 45, p = 0.08*
Hours a day (weekend) video games	2.04 ± 1.25 (7)	5,25 ± 2.06 (4)	*t* = *−3.256, df = 9, p = 0.04*
Having holidays (2 times or more per year)	62.2	33.3	FET *p* = 0.104

In italics, comparisons which reached significance before applying Bonferroni correction. In bold, comparisons which remained significant after Bonferroni correction.

Table 5 summarizes parental responses to an *ad hoc* questionnaire about the presence of a putative addiction to new technologies (including IGD). Several of the questions were statistically related to the IGD diagnosis. The first question evaluating the use of NT (*Do you think your child has an addiction to any of the following technologies?*) was affirmatively answered by all parents of children and adolescents with ADHD who were displaying IGD.

**Table 5:** *Ad hoc* questionnaire about a putative ANT (parents).

Item	No VG addiction *n* (%)	VG addiction *n* (%)	Significance
1. Do you think your child has an addiction to any of the following technologies? (Yes)	27 (71.1)	13 (100)	*FET p = 0.046*
2. Has your child gotten the urge to use new technologies (smartphone, video games, or the internet) to relieve tension, relax, or decrease psychological distress in the past year? (Yes)	13 (38.2)	7 (70)	FET *p* = 0.147
3. Has your child been using new technologies more frequently or for longer duration than initially planned? (Yes)	20 (58.8)	8 (80)	FET *p* = 0.283
4. Does your child have a persistent desire to quit the use of new technologies, but is unable to stop? (Yes)	14 (41.2)	9 (90)	*FET p = 0.010*
5. Has your child ever missed or reduced a social engagement, work, school, or other recreational activities because he/she was involved in activities related to new technologies? (Yes)	14 (41.2)	6 (60)	FET *p* = 0.472
6. Does your child continue using new technologies despite knowing the problems related to its use? (Yes)	23 (67.6)	10 (100)	*FET p = 0.046*
7. Has your child tried to stop using new technologies, but is unable to do so or it took him/her a lot of effort? (Yes)	18 (52.9)	9 (90)	FET *p* = 0.062
8. Do you feel that your child needs to spend more and more time on new technologies in order to feel good, less anxious, or emotionally fine? (Yes)	11 (32.4)	8 (80)	*FET p = 0.011*
9. Does your child feel a strong desire to use new technologies even without any particular reason? (Yes)	18 (52.9)	10 (100)	*FET p = 0.007*
10. Has your child gotten into trouble at school/work/home due to new technologies? (Yes)	14 (42.2)	4 (40)	FET *p* = 1
11. Does your child use new technologies in situations in which it is physically hazardous? (i.e., crossing a road while using smartphone) (Yes)	6 (17.6)	4 (40)	FET *p* = 0.199
12. Does your child feel bad, anxious or annoyed when he/she wishes to use new technologies but cannot do so at the time? (Yes)	17 (50)	10 (100)	*FET p = 0.004*

In italics, comparisons which reached significance before applying Bonferroni correction. In bold, comparisons which remained significant after Bonferroni correction.

Finally, Table 6 displays the relationship between IGD status and a set of academic performance measures. We found not a single statistically significant association.

**Table 6:** Academic impact of IGD.

	IGD (*n* = 38) %	IGD (*n* = 13) %	Significance
Repeating at least one school year	25	30.8	FET *p* = 0.723
Teaching support at school (Yes)	34.3	41.7	FET *p* = 0.773
Adapting early childhood curriculum at school (Yes)	28.6	41.7	FET *p* = 0.481
Teaching support at home (Yes)	40	41.7	FET *p* = 1

In italics, comparisons which reached significance before applying Bonferroni correction. In bold, comparisons which remained significant after Bonferroni correction.

## Discussion

The present study reports several interesting findings that may help in delineating a specific profile for those children and adolescents with ADHD who display an IGD disorder. Interestingly, we found no significant socio-demographic and neurodevelopmental differences between ADHD patients with and without IGD, with the single exception of age of walking (first steps), which was attained first by children and adolescents with ADHD and IGD. As expected, we also found a relationship between the hyperactivity subtype and IGD, which was further supported by the clinical scales and some objective measures of the CPT-3. Even more interesting, and in some sense, intriguing, we unexpectedly found that patients with ADHD and IGD displayed lower levels of blood ferritin and vitamin D. Another interesting finding has to do with the pattern of gaming use and related issues. Thus, we found that children with ADHD and IGD had a first contact with the internet at a statistically significantly younger age than those without IGD. Furthermore, they spend more hours a day (both on school days and weekends) playing video games, but these results must be interpreted cautiously given the small proportion of patients answering these questions. But the most relevant finding was indeed a negative, unexpected result: contrary to our thinking, we found that children with ADHD and IGD did not have a statistically significantly worse academic performance than those without IGD.

The first interesting finding of the present study is the lack of association of most socio-demographic and clinical data with the presence of IGD, giving further relevance to the few associations reported. Thus, our findings suggest that children and adolescents with ADHD and with or without IGD are not very different in terms of sociodemographic, neurodevelopmental, and ADHD characteristics. However, compared with children and adolescents with ADHD but not IGD, ADHD children and adolescents with ADHD and IGD: (1) began walking at a younger age; (2) had predominantly hyperactive/impulsive type (ADHD/HI) or combined type (ADHD/C) ADHD, using converging data coming from clinical data, scales (higher scores on the hyperactivity part of the SNAP-IV scale), and objective measures (higher scores only in commissions, a measure of impulsivity). This last finding is in keeping with the literature, as some have suggested that impulsivity explains the relationship between ADHD and IGD (17, 27); (3) had a different pattern of gaming, characterized by a lower age at first contact with the internet (but not with video games), and spent more hours on video games both during school days and weekends. Something which is worrisome is that all children and adolescents included in this study reported having gotten their first contact with new technologies (either smartphones, video games, or the internet) before they were 9 years old. Guided by the concern surrounding the way children use smartphone technologies and the internet, the European Commission conducted a study under the program “Strategies for a Better Internet for Children,” which started in May 2012 (28). The authors reported that “the average age of first contact with information technologies was around 9 years.” On the other hand, it was also expected to find that IGD patients would spend more time playing video games. Video games provide quick, immediate rewards, and an artificial living environment where children and adolescents can escape from daily problems (29). Furthermore, a recent meta-analysis reported that gaming time was one risk factor for IGD (30). In any case, the patients with IGD in our study averaged 18.85 h of gaming a week, clearly below the average time for disordered gaming of 34.53 h of gaming a week within the APA framework (31).

Another interesting finding was the association between low ferritin (marginally statistically non-significant, but clearly of clinical relevance) and vitamin D with IGD status. Regarding serum ferritin, the relationship between low blood ferritin levels and ADHD is controversial, with positive and negative studies (32–34). Furthermore, serum ferritin has been inversely correlated with baseline inattention, and hyperactivity/impulsivity (35). Regarding vitamin D, the low blood vitamin D in our IGD patients could be explained by the fact that, by spending more time playing video games, they could be spending less time doing outdoor activities, with less sun exposure. A recent meta-analysis of observational studies concluded that there is an inverse association between serum 25(OH)D and ADHD in young patients (36). Furthermore, low serum vitamin D has also been related to impulsivity in patients with eating disorders (37). These studies give support to vitamin D supplementation to vulnerable populations (38). However, in a mixed-design study using both human and mice data, the authors concluded that higher anxiety level, hyperactivity level and aggressiveness were reported both in participants with either low or high vitamin D level, suggesting a “U” relationship between serum vitamin D concentration and behavior, emotion, and attention (39).

Finally, another interesting result is that the *ad hoc* questionnaire that we used to evaluate a putative addiction to new technologies performed well in discriminating which patients have IGD. Indeed, 100% of the parents of those children and adolescents with ADHD and IGD as measured by the ADITEC-V were rated as having an addiction to new technologies. In addition, item 10 (*Has your child gotten into trouble at school/work/home due to new technologies?*) was not associated with IGD status. This finding was in keeping with the most interesting finding of the present study: a lack of association between IGD and academic performance. *A priori*, and based on the literature suggesting that excessive gaming may be associated with decreased academic achievement (40–43), we expected to find such a relationship. However, our findings are more in keeping with the negative results reported in a study of 228 medical students (44). The authors found the classic association between male gender and IGD, but did not find a relationship between IGD and academic achievement. Some studies suggest that the negative relationship between IGD and academic achievement may be mediated by academic engagement: dedicating much time to video games takes time away from studying. However, children with ADHD tend to show lower levels of academic engagement (45, 46) regardless of whether or not they suffer IGD. This lack of difference between patients with ADHD with or without IGD in academic performance is likely due to the small sample size, but it is also possible that children with ADHD would tend to avoid doing homework and studying, be it through problematic use of video games or some other way.

Furthermore, as we suggest in a recent quasi-systematic review, video games can be either angels or demons (47). Indeed, several recent studies suggest that video games may help children and adolescents with or without ADHD in improving poor social skills. Although the influence of video games on children’s mental health is usually negatively perceived, a recent study with 3,195 children aged 6–11 years old reported that high video game use was associated with 1.75 times the odds of high intellectual functioning, and a decreased risk of peer relationship problems (48). Moreover, a randomized trial with 69 children aged 7–11 years old with poor social skills demonstrated that those children assigned to treatment with an interactive online adventure game for 9 weeks improved significantly more than the waiting list controls “in social literacy, social anxiety, bullying victimization, and social satisfaction” (49). In addition, playing video games increases synaptic dopamine, and “internet video game playing might be a means of self-medication for children with ADHD” (50). However, a meta-analysis of 101 studies (51) showed negative effects, although minimal, of video games on academic performance, prosocial behavior, and aggression.

Some limitations should be mentioned. The main limitation is the small sample sizes and the subsequent low statistical power. Conclusions regarding lack of differences should be taken cautiously. In turn, the differences which remained significant despite the small sample size and the Bonferroni corrections are presumably quite large, but their generalization beyond samples similar to ours is not guaranteed. Also, there are some other limitations which should be taken into account. First, our study is cross-sectional. Accordingly, bias may affect our results, and establishing causal links is not possible, being confronted with the classic chicken and egg dilemma. Secondly, the small sample size meant may have compromised some results that did not reach statistical significance. Thirdly, we based our IGD diagnosis on the 85% cut-off point of a relatively new questionnaire only available in Spanish, the ADITEC-V. However, the ADITEC-V correlated with item 10 of the *ad hoc* questionnaire, and all results are very consistent. Finally, the use of an *ad hoc* questionnaire (based on modified DSM-IV-TR criteria for substance dependence) for parents to evaluate their perceptions of the use of video games by their children is a strategy that has been used for evaluating other behavioral addictions, such as tanning (52, 53).

## Conclusion

Most parents of children and adolescents with ADHD were concerned that their children may have an IGD. However, only 25% met the criteria for IGD used in our study. Interestingly, our study strongly suggests that IGD was associated with several risk factors that focus on the relationship between the hyperactivity-impulsivity domain of ADHD and IGD from different and converging perspectives. Regarding video gaming time, we found that children and adolescents with ADHD and IGD had a particular gaming profile characterized by a first contact with the internet at a younger age, and a more abusive pattern of use of video games. Another interesting finding was that patients with ADHD and IGD displayed lower levels of blood ferritin and vitamin D. Finally, we found that children with ADHD and IGD did not have a statistically significantly worse academic performance than those without IGD. Indeed, estimates for the length that IGD can last vary widely, but the reasons behind it are unclear (54).

Future directions for research may include, among others: (1) the development of randomized controlled trials for treating IGD in patients with (or without) ADHD; (2) the study of social adjustment as a protective factor against developing IGD; (3) the study of serious and non-serious video games in the development of an IGD; and (4) the study of how some characteristics of video games (i.e., gaming time, frequency of play, etc.) interact with clinical and neuropsychological characteristics of children and adolescents with ADHD. Additional research is clearly needed on IGD.

## Data availability statement

The original contributions presented in the study are included in the article/supplementary materials, further inquiries can be directed to the corresponding authors.

## Ethics statement

The studies involving human participants were reviewed and approved by Institutional Review Board of the Puerta de Hierro University Hospital-Majadahonda. Written informed consent to participate in this study was provided by the participants’ legal guardian/next of kin.

## Author contributions

All authors have participated sufficiently in the work to take public responsibility for the content. The corresponding author affirms that he had access to all data from the study, both what is reported and what is unreported, and also had complete freedom to direct its analysis and reporting. HB-F conceived and designed the study. ÁM-G and HB-F gathered all clinical and protocol data, and designed the database and entered most of the data. HB-F and MB-F are responsible for data analyses, literature searches, draft and revision of the manuscript’s initial versions. FS-S provided critical information on the concept of video game addiction (VGA), and in particular, on the use of appropriate cut-off points of the ADITEC-V test. All authors reviewed the manuscript and provided conceptual guidance for improving the study. All authors read, critically revised and approved the final version of the manuscript; no other potential authors have been omitted from authorship.

## Conflict of interest

In the last 24 months, HB-F has received lecture fees from Takeda and Rubio. He is the principal investigator (PI) of an iPFIS (Contratos Predoctorales de Formación e Investigación en Salud) research contract (Instituto de Salud Carlos III; IFI16/00039) and the co-PI of a MINECO research grant (RTI2018-101857-B-I00); he is the recipient of (1) a grant from the Fundación para la Innovación y la Prospectiva en Salud en España and (2) an IDIPHIPSA intensification grant; involved in 2 clinical trials (MENSIA KOALA, NEWROFEED Study; ESKETSUI2002); Founder of Haglaia Solutions. He is also an employee and member of the advisory board of Ita Salud Mental (Korian). He is the member of a consortium developing a serious videogame for treating ADHD called “The Secret Trail of Moon” (TSTM) -still non-commercialized-. FS-S was employed by company TEA Ediciones.

The remaining authors declare that the research was conducted in the absence of any commercial or financial relationships that could be construed as a potential conflict of interest.

## Publisher’s note

All claims expressed in this article are solely those of the authors and do not necessarily represent those of their affiliated organizations, or those of the publisher, the editors and the reviewers. Any product that may be evaluated in this article, or claim that may be made by its manufacturer, is not guaranteed or endorsed by the publisher.

## References

[1] Weiss MD Baer S Allan BA Saran K Schibuk H. The screens culture: impact on ADHD. Atten Defic Hyperact Disord. (2011) 3:327–4. doi: 10.1007/s12402-011-0065-z, PMID: 21948003 PMC3220824

[2] Choliz M. Mobile phone addiction: a point of issue. Addiction. (2010) 105:373–4. doi: 10.1111/j.1360-0443.2009.02854.x 20078493

[3] Osborne LA Romano M Re F Roaro A Truzoli R Reed P. Evidence for an internet addiction disorder: internet exposure reinforces color preference in withdrawn problem users. J Clin Psychiatry. (2016) 77:269–4. doi: 10.4088/JCP.15m10073 26930524

[4] Rehbein F Kliem S Baier D Mossle T Petry NM. Prevalence of internet gaming disorder in German adolescents: diagnostic contribution of the nine DSM-5 criteria in a state-wide representative sample. Addiction. (2015) 110:842–1. doi: 10.1111/add.12849, PMID: 25598040

[5] Bargeron AH Hormes JM. Psychosocial correlates of internet gaming disorder: psychopathology, life satisfaction, and impulsivity. Comput Hum Behav. (2017) 68:388–4. doi: 10.1016/j.chb.2016.11.029

[6] Milani L la Torre G Fiore M Grumi S Gentile DA Ferrante M. Internet gaming addiction in adolescence: risk factors and maladjustment correlates. Int J Ment Heal Addict. (2018) 16:888–4. doi: 10.1007/s11469-017-9750-2

[7] Rehbein F Baier D. Family-, media-, and school-related risk factors of video game addiction. J Media Psychol. (2013) 25:118–8. doi: 10.1027/1864-1105/a000093

[8] Weinstein A Yaacov Y Manning M Danon P Weizman A. Internet addiction and attention deficit hyperactivity disorder among schoolchildren. Isr Med Assoc J. (2015) 17:731–4. PMID: 26897972

[9] Polanczyk G de Lima MS Horta BL Biederman J Rohde LA Polanczyk GV. The worldwide prevalence of ADHD: a systematic review and metaregression analysis annual research review: a meta-analysis of the worldwide prevalence of mental disorders in children and adolescents. Am J Psychiatry. (2007) 164:942–8. doi: 10.1111/jcpp.12381 17541055

[10] Marmet S Studer J Grazioli VS Gmel G. Bidirectional associations between self-reported gaming disorder and adult attention deficit hyperactivity disorder: evidence from a sample of Young Swiss men. Front Psych. (2018) 9:649. doi: 10.3389/fpsyt.2018.00649, PMID: 30618855 PMC6297670

[11] Barkley RA. Behavioral inhibition, sustained attention, and executive functions: constructing a unifying theory of ADHD. Psychol Bull. (1997) 121:65–94. doi: 10.1037/0033-2909.121.1.65, PMID: 9000892

[12] Barkley RA. Attention-deficit hyperactivity disorder: A handbook for diagnosis and treatment. Emotional dysregulation is a core component of ADHD. ed. Barkley R. A. The Guilford Press (2015):81–115.

[13] Özbaran B Kalyoncu T Köse S. Theory of mind and emotion regulation difficulties in children with ADHD. Psychiatry Res. (2018) 270:117–2. doi: 10.1016/j.psychres.2018.09.034, PMID: 30245374

[14] Bioulac S Arfi L Bouvard MP. Attention deficit/hyperactivity disorder and video games: a comparative study of hyperactive and control children. Eur Psychiatry. (2008) 23:134–1. doi: 10.1016/j.eurpsy.2007.11.002, PMID: 18206354

[15] Berloffa S Salvati A D'Acunto G Fantozzi P Inguaggiato E Lenzi F. Internet gaming disorder in children and adolescents with attention deficit hyperactivity disorder. Children (Basel). (2022) 9:1–11. doi: 10.3390/children9030428 PMC8947564 35327800

[16] Lee J Bae S Kim BN Han DH. Impact of attention-deficit/hyperactivity disorder comorbidity on longitudinal course in internet gaming disorder: a 3-year clinical cohort study. J Child Psychol Psychiatry. (2021) 62:1110–9. doi: 10.1111/jcpp.13380, PMID: 33751554

[17] Menéndez-García A Jiménez-Arroyo A Rodrigo-Yanguas M Marin-Vila M Sánchez-Sánchez F Roman-Riechmann E. Internet, video game and mobile phone addiction in children and adolescents diagnosed with ADHD: a case-control study. Adicciones. (2022) 34:208. doi: 10.20882/adicciones.1469, PMID: 33338245

[18] Chóliz M Marco C Chóliz C. ADITEC. evaluación y prevención de la adicción a internet, móvil y videojuegos. Madrid: TEA Ediciones (2016).

[19] Swanson JM. SNAP-IV scale. Irvine: University of California Child Development Center (1995).

[20] Grañana N Richaudeau A Gorriti CR O'Flaherty M Scotti ME Sixto L. Assessment of attention deficit hyperactivity: SNAP-IV scale adapted to Argentina. Rev Panam Salud Publica. (2011) 29:344–9. doi: 10.1590/S1020-49892011000500007, PMID: 21709939

[21] Conners C. Conners abbreviated symptom questionnaire: Parent version, teacher version manual. Toronto: Multi-Health Systems (1990).

[22] Ullmann RK Sleator EK Sprague RL. A change of mind: the Conners abbreviated rating scales reconsidered. J Abnorm Child Psychol. (1985) 13:553–65. doi: 10.1007/BF00923141, PMID: 4078186

[23] Gioia GA Isquith PK Guy SC Kenworthy L. Behavior rating inventory of executive function. Odessa, FL: Psychological Assessment Resources (2000).

[24] Mahone EM Cirino PT Cutting LE Cerrone PM Hagelthorn KM Hiemenz JR. Validity of the behavior rating inventory of executive function in children with ADHD and/or Tourette syndrome. Arch Clin Neuropsychol. (2002) 17:643–62. doi: 10.1093/arclin/17.7.643 14591848

[25] Conners CK. Conners continuous performance test- third edition (Conners CPT 3) & Conners continuous auditory test of attention (Conners CATA): Technical manual. New York: Multi-Health Systems Inc (2014).

[26] Wechsler D. Wechsler intelligence scale for children. Fourth edition (WISC-IV) ed: [Database record] (2003).

[27] Haghbin M Shaterian F Hosseinzadeh D Griffiths MD. A brief report on the relationship between self-control, video game addiction and academic achievement in normal and ADHD students. J Behav Addict. (2013) 2:239–43. doi: 10.1556/JBA.2.2013.4.7, PMID: 25215206 PMC4154570

[28] Vismara MFM Toaff J Pulvirenti G Settanni C Colao E Lavano SM. Internet use and access, behavior, Cyberbullying, and grooming: results of an investigative whole City survey of adolescents. Interact J Med Res. (2017) 6:e9. doi: 10.2196/ijmr.6231, PMID: 28851675 PMC5596301

[29] Gentile DA Swing EL Lim CG Khoo A. Video game playing, attention problems, and impulsiveness: evidence of bidirectional causality psychology of popular media. Culture. (2012) 1:62–70. doi: 10.1037/a0026969

[30] Ropovik I Martončik M Babinčák P Baník G Vargová L Adamkovič M. Risk and protective factors for (internet) gaming disorder: a meta-analysis of pre-COVID studies. Addict Behav. (2022) 139:107590. doi: 10.1016/j.addbeh.2022.107590 36571943

[31] Pontes HM Schivinski B Kannen C Montag C. The interplay between time spent gaming and disordered gaming: a large-scale world-wide study. Soc Sci Med. (2022) 296:114721. doi: 10.1016/j.socscimed.2022.114721 35168056

[32] Donfrancesco R Parisi P Vanacore N Martines F Sargentini V Cortese S. Iron and ADHD: time to move beyond serum ferritin levels. J Atten Disord. (2013) 17:347–7. doi: 10.1177/1087054711430712 22290693

[33] Tang CY Wen F. Serum ferritin levels in children with attention deficit hyperactivity disorder and tic disorder. World J Clin Cases. (2022) 10:7749–59. doi: 10.12998/wjcc.v10.i22.7749, PMID: 36158507 PMC9372851

[34] Oner P Oner O Azik FM Cop E Munir KM. Ferritin and hyperactivity ratings in attention deficit hyperactivity disorder. Pediatr Int. (2012) 54:688–2. doi: 10.1111/j.1442-200X.2012.03664.x, PMID: 22591427 PMC4677666

[35] Calarge C Farmer C DiSilvestro R Arnold LE. Serum ferritin and amphetamine response in youth with attention-deficit/hyperactivity disorder. J Child Adolesc Psychopharmacol. (2010) 20:495–2. doi: 10.1089/cap.2010.0053, PMID: 21186968 PMC3003494

[36] Kotsi E Perrea DN. Vitamin D levels in children and adolescents with attention-deficit hyperactivity disorder (ADHD): a meta-analysis. Atten Defic Hyperact Disord. (2019) 11:221–2. doi: 10.1007/s12402-018-0276-7, PMID: 30367389

[37] Todisco P Meneguzzo P Vogazianos P Garolla A Antoniades A Tozzi F. Relation between vitamin D and impulse behaviours in patients with eating disorder: a pilot observational study. Eur Eat Disord Rev. (2020) 28:587–3. doi: 10.1002/erv.2740, PMID: 32372472

[38] Hemamy M Heidari-Beni M Askari G Karahmadi M Maracy M. Effect of vitamin D and magnesium supplementation on behavior problems in children with attention-deficit hyperactivity disorder. Int J Prev Med. (2020) 11:4. doi: 10.4103/ijpvm.IJPVM_546_17 32089804 PMC7011463

[39] Wang X Jiao X Xu M Wang B Li J Yang F. Effects of circulating vitamin D concentrations on emotion, behavior and attention: a cross-sectional study in preschool children with follow-up behavior experiments in juvenile mice. J Affect Disord. (2020) 275:290–8. doi: 10.1016/j.jad.2020.06.043, PMID: 32734921

[40] Hawi NS Samaha M Griffiths MD. Internet gaming disorder in Lebanon: relationships with age, sleep habits, and academic achievement. J Behav Addict. (2018) 7:70–8. doi: 10.1556/2006.7.2018.16, PMID: 29486571 PMC6035028

[41] Samaha M Hawi N. Internet gaming disorder and its relationships with student engagement and academic performance. Res Anthol Game Design Dev Usage Soc Impact. (2023):1704–20.

[42] Wartberg L Zieglmeier M Kammerl R. Accordance of adolescent and parental ratings of internet gaming disorder and their associations with psychosocial aspects. Cyberpsychol Behav Soc Netw. (2019) 22:264–08. doi: 10.1089/cyber.2018.0456, PMID: 30801222

[43] Burgess SR Stermer SP Burgess MC. Video game playing and academic performance in college students. Coll Stud J. (2012) 46:376–8.

[44] Al Asqah MI Al Orainey AI Shukr MA Al Oraini HM Al Turki YA. The prevalence of internet gaming disorder among medical students at King Saud University, Riyadh, Saudi Arabia. A cross-sectional study Saudi Med J. (2020) 41:1359–63. doi: 10.15537/smj.2020.12.05584, PMID: 33294895 PMC7841580

[45] Major A. Sources of self-efficacy, self-efficacy for self-regulated learning, and student engagement in adolescents with ADHD. Toronto: University of Toronto (Canada) (2016).

[46] Junod REV DuPaul GJ Jitendra AK Volpe RJ Cleary KS. Classroom observations of students with and without ADHD: differences across types of engagement. J Sch Psychol. (2006) 44:87–4. doi: 10.1016/j.jsp.2005.12.004

[47] Rodrigo-Yanguas M González-Tardón C Bella-Fernández M Blasco-Fontecilla H. Serious video games: angels or demons in patients with attention-deficit hyperactivity disorder? A quasi-systematic review. Front Psychiatry. (2022) 13:798480. doi: 10.3389/fpsyt.2022.798480, PMID: 35573357 PMC9091561

[48] Kovess-Masfety V Keyes K Hamilton A Hanson G Bitfoi A Golitz D. Is time spent playing video games associated with mental health, cognitive and social skills in young children? Soc Psychiatry Psychiatr Epidemiol. (2016) 51:349–7. doi: 10.1007/s00127-016-1179-6, PMID: 26846228 PMC4814321

[49] Sanchez R Brown E Kocher K DeRosier M. Improving Children's mental health with a digital social skills development game: a randomized controlled efficacy trial of adventures aboard the S.S. GRIN Games Health J. (2017) 6:19–27. doi: 10.1089/g4h.2015.0108, PMID: 28051877

[50] Han DH Lee YS Na C Ahn JY Chung US Daniels MA. Is time spent playing video games associated with mental health, cognitive and social skills in young children?, The effect of methylphenidate on internet video game play in children with attention-deficit/hyperactivity disorder. Compr Psychiatry. (2009) 50:251–6. doi: 10.1016/j.comppsych.2008.08.011, PMID: 19374970

[51] Ferguson CJ. Do angry birds make for angry children? A meta-analysis of video game influences on children’s and adolescents’ aggression, mental health, prosocial behavior, and academic performance. Perspect Psychol Sci. (2015) 10:646–6. doi: 10.1177/1745691615592234, PMID: 26386002

[52] Kourosh AS Harrington CR Adinoff B. Tanning as a behavioral addiction. Am J Drug Alcohol Abuse. (2010) 36:284–08. doi: 10.3109/00952990.2010.491883 20545604

[53] Blasco-Fontecilla H Artieda-Urrutia P Berenguer-Elias N Garcia-Vega JM Fernandez-Rodriguez M. Is time spent playing video games associated with mental health, cognitive and social skills in young children?, Are major repeater patients addicted to suicidal behavior? Adicciones. (2014) 26:321–3. doi: 10.20882/adicciones.38, PMID: 25580865

[54] Gentile DA Bailey K Bavelier D Brockmyer JF Cash H Coyne SM, , . Is time spent playing video games associated with mental health, cognitive and social skills in young children? Internet gaming disorder in children and adolescents Pediatrics (2017). 140:S81–S85, doi: 10.1542/peds.2016-1758H 29093038

